# Outcomes after surgery in patients with and without recent influenza: a nationwide population-based study

**DOI:** 10.3389/fmed.2023.1117885

**Published:** 2023-06-09

**Authors:** Fai Lam, Chien-Chang Liao, Ta-Liang Chen, Yu-Min Huang, Yuarn-Jang Lee, Hung-Yi Chiou

**Affiliations:** ^1^School of Public Health, College of Public Health, Taipei Medical University, Taipei, Taiwan; ^2^Department of Anesthesiology, Taipei Medical University Hospital, Taipei, Taiwan; ^3^Department of Anesthesiology, School of Medicine, College of Medicine, Taipei Medical University, Taipei, Taiwan; ^4^Anesthesiology and Health Policy Research Center, Taipei Medical University Hospital, Taipei, Taiwan; ^5^Research Center of Big Data and Meta-Analysis, Wan Fang Hospital, Taipei Medical University, Taipei, Taiwan; ^6^School of Chinese Medicine, College of Chinese Medicine, China Medical University, Taichung, Taiwan; ^7^Department of Anesthesiology, Wan Fang Hospital, Taipei Medical University, Taipei, Taiwan; ^8^Department of Surgery, School of Medicine, College of Medicine, Taipei Medical University, Taipei, Taiwan; ^9^Division of Gastrointestinal Surgery, Department of Surgery, Taipei Medical University Hospital, Taipei Medical University, Taipei, Taiwan; ^10^Division of Infectious Disease, Department of Internal Medicine, Shuang Ho Hospital, Taipei Medical University, New Taipei, Taiwan; ^11^Institute of Population Health Sciences, National Health Research Institutes, Miaoli, Taiwan

**Keywords:** surgery, infectious diseases, mortality, perioperative outcomes, influenza

## Abstract

**Background:**

The influence of recent influenza infection on perioperative outcomes is not completely understood.

**Method:**

Using Taiwan’s National Health Insurance Research Data from 2008 to 2013, we conducted a surgical cohort study, which included 20,544 matched patients with a recent history of influenza and 10,272 matched patients without. The main outcomes were postoperative complications and mortality. We calculated odds ratios (ORs) and 95% confidence intervals (CIs) for the complications and for mortality in patients with a history of influenza within 1–14 days or 15–30 days compared with non-influenza controls.

**Results:**

Compared with patients who had no influenza, patients with influenza within preoperative days 1–7 had increased risks of postoperative pneumonia (OR 2.22, 95% CI 1.81–2.73), septicemia (OR 1.98, 95% CI 1.70–2.31), acute renal failure (OR 2.10, 95% CI 1.47–3.00), and urinary tract infection (OR 1.45, 95% CI 1.23–1.70). An increased risk of intensive care admission, prolonged length of stay, and higher medical expenditure was noted in patients with history of influenza within 1–14 days.

**Conclusion:**

We found that there was an association between influenza within 14 days preoperatively and the increased risk of postoperative complications, particularly with the occurrence of influenza within 7 days prior to surgery.

## Introduction

Influenza is a viral infection that causes morbidity and mortality, particularly in children, older populations, and those with chronic diseases. In 2018, a modeling study estimated that global influenza-associated respiratory mortality is between 290,000 to 650,000 annually (1), which is higher than that previously reported from the World Health Organization (250,000 to 500,000 annually) in 2016 (2). Influenza causes loss of life and heavy medical resource utilization, including approximately 61,100 lost life-years, 3.1 million hospital days, 31.4 million outpatient visits, $10.4 billion United States dollars (USD) in healthcare costs, and $16.3 billion USD in lost earnings associated with influenza complications in the United States (3). The estimated annual losses due to influenza were approximately $500 billion (USD), or 0.6% of the global income (4).

Influenza may lead to acute and profound immune responses in the respiratory tract. People infected with influenza have increased rates of complications and higher mortality, particularly those with chronic medical conditions, such as heart failure (5), chronic obstructive pulmonary disease (6), and end-stage renal disease (7). In addition, growing evidence has shown that influenza may be a trigger of acute myocardial infarction and stroke (8–10).

At least 312.9 million surgical procedures are performed annually worldwide, and this number is increasing (11). Postoperative complications and mortality are a substantial global public-health concern. In addition, it is possible that patients infected with influenza will also undergo surgery. However, limited data are available on outcomes after surgery in patients with a recent history of influenza. Some studies have reported that children with perioperative upper respiratory tract infections had increased postoperative respiratory and other complications after undergoing cardiac surgery (12, 13). It also has been reported that the risks of postoperative respiratory and cardiovascular complications are increased in adults with a recent respiratory infection (14, 15).

The optimal timing of elective surgery in patients with a recent history of influenza remains unclear and the influence of influenza on postoperative outcomes has not been fully studied. In the current practice guidelines of the Centers for Disease Control in Taiwan, there is no recommendation about patients with influenza before surgery. The policy of postponing surgery for patients with recent influenza has not been fully discussed and there are no related practice guidelines. Therefore, we conducted a real-world analysis, based on an insurance database to investigate possible adverse outcomes after major surgery in patients with a recent history of influenza. The aim of this study was to evaluate the postoperative complications and mortality in patients with and without recent influenza before surgery. Our hypothesis is that patients who had recent influenza before surgery may have more postoperative complications and higher postoperative mortality compared with surgical patients without influenza.

## Methods

### Source of data

In this study, we used research data from the national health insurance database in Taiwan, where more than 99% of 23 million people are covered by insurance. National health insurance is administered by the government, and we utilized a database that included demographic characteristics, physicians’ primary and secondary diagnoses, treatment procedures, prescriptions, and medical expenditures. Many scientific articles, based on this same research data, have been accepted by prominent journals worldwide for publication (16, 17).

### Availability of data and materials

The data that support the findings of this study are available from the Ministry of Health and Welfare, Taiwan, but restrictions apply to the availability of these data, which were used under license for the current study, and so are not publicly available. Data are however available from the authors upon reasonable request and with permission of the Ministry of Health and Welfare, Taiwan. Researchers could contact the liaison and make application for the database using the following information: https://dep.mohw.gov.tw/dos/cp-2516-59203-113.html.

### Ethical approval

According to the regulations of the Ministry of Health and Welfare, the electronic database was decoded and patient identifiers scrambled for further research access; therefore, the personal privacy of patients was protected and informed consent was not required. Our study waived the need of informed consent and it was approved by the institutional review board of Taipei Medical University (TMU-JIRB-202302005; TMU-JIRB-201910032; TMU-JIRB-201905042; TMU-JIRB-201902053). All methods were carried out in accordance with relevant guidelines and regulations. All experimental protocols were approved by the Institutional Review Board of Taipei Medical University.

### Study design

Taiwan’s National Health Insurance Research Database is one of the large health care administrative databases in the world (18). In this study, we defined the current surgical cohort as all insured people who underwent major surgery in 2008–2013. From the health insurance research database, we identified 3,182,869 adults aged more than 18 years who underwent major surgery between 2008 and 2013. Of these, 28,139 were infected with influenza within 30 days prior to surgery. Supplementary Figure S1 shows the selection of study patients. This real-world analysis with propensity-score matching was conducted to balance the baseline characteristics of the three groups, including patients with influenza within 15–30 days preoperatively, patients with influenza within 1–14 days preoperatively, and a control group without preoperative influenza. Patients were matched in a 1:1:1 ratio, and age, low-income status, whether the operation took place in a medical center, coexisting medical conditions, types of surgery, and types of anesthesia were all considered in our propensity-score matching procedure.

### Measures and definitions

We defined major surgery as surgical procedures requiring general, epidural, or spinal anesthesia as well as hospitalization for more than 1 day. All surgical patients who received major surgery were included in this study. The low-income status of patients was defined as those qualifying for waived medical copayments by criteria from the Ministry of Health and Welfare. Based on previous reports, the levels of surgical risk were categorized into low-risk, medium-risk, high-risk, heart surgery, and other (19, 20).

We used physician diagnosis and the International Classification of Diseases, Ninth Revision, Clinical Modification (ICD-9-CM) to identify influenza, preoperative medical diseases, and postoperative complications. Based on our previous studies, preoperative medical diseases were determined from the 24-month preoperative period and included hypertension (ICD-9-CM 401–405), mental disorders (ICD-9-CM 290–319), diabetes (ICD-9-CM 250), ischemic heart disease (ICD-9-CM 410–414), chronic obstructive pulmonary disease (ICD-9-CM 491,492, 496), hyperlipidemia (ICD-9-CM 272.0, 272.1, 272.2, 272.4), liver cirrhosis (ICD-9-CM 571.2, 571.5, 571.6), and heart failure (ICD-9-CM 428). Renal dialysis was defined by administration codes (D8 and D9). The primary outcome was the 30-day in-hospital mortality following the index surgery for patients with and without influenza. Postoperative complications included postoperative bleeding (ICD-9-CM codes 998.0, 998.1, and 998.2), pneumonia (ICD-9-CM codes 480–486), septicemia (ICD-9-CM codes 038 and 998.5), urinary tract infection (ICD-9-CM code 599.0), deep wound infection (ICD-9-CM code 958.3), pulmonary embolism (ICD-9-CM code 415), acute renal failure (ICD-9-CM code 584), acute myocardial infarction (ICD-9-CM code 410), and stroke (ICD-9-CM codes 430–438). We also compared admission to intensive care, length of hospital stay, and medical expenditures following the index surgery for patients with and without influenza in the first 30-day postoperative period. According to the suggestions from the previous study (20), we considered postoperative pneumonia, septicemia, urinary tract infection, and acute renal failure as postoperative adverse events in this study.

### Statistical analyses

The propensity-score matched-pair analysis was used to determine the association between influenza and our primary outcome (mortality after surgery). We developed a non-parsimonious, multivariable, logistic regression model to estimate a propensity score for the surgical patients with influenza. We used a propensity-score matched-pair design combined with frequency matching to balance the distribution of the covariates, including age, sex, low-income status, type of surgery, type of anesthesia, level of surgical risk, hypertension, mental disorders, diabetes, ischemic heart disease, chronic obstructive pulmonary disease, hyperlipidemia, liver cirrhosis, heart failure, and renal dialysis between surgical patients with and without influenza. For achieving a balance of covariates within matched pairs, we performed a structured, iterative approach to refine this logistic regression model using a 1:1 case–control match on the propensity score. We then matched (without replacement) patients who had influenza with those who did not by using a greedy matching algorithm. The algorithm proceeds sequentially to the lowest digit match on propensity score (1 digit). This is referred to as the 8–1 digit match.

To describe baseline characteristics, we expressed continuous variables as the mean ± standard deviation by using t-tests. The categorical variables are expressed as numbers (percentage) from using chi-square tests. We used logistic regression models to assess the risks of postoperative complications and mortality associated with influenza by calculating adjusted odds ratios (CIs) and 95% confidence intervals (CIs). We also performed subgroup analyses and evaluated the risks of pneumonia, septicemia, and urinary tract infection in patients with a recent history of influenza within 1–7, 8–14, or 15–30 days prior to surgery.

## Results

Among 30,816 eligible surgical patients (Table 1), the results of matching by propensity score revealed no significant differences in age, sex, low-income status, type of surgery, type of anesthesia, level of surgical risk, or in the presence of hypertension, mental disorders, diabetes, ischemic heart disease, chronic obstructive pulmonary disease, hyperlipidemia, liver cirrhosis, heart failure, or renal dialysis among the three groups (no influenza, influenza within 1–14 days, and influenza within 15–30 days). Supplementary Table S1 shows the characteristics of surgical patients with and without influenza before matching.

**Table 1:** Characteristics of surgical patients with and without influenza (after matching).

	No influenza (*N* = 10,272)	Influenza 15–30 days (*N* = 10,272)	Influenza 1–14 days (*N* = 10,272)	Value of *p*
Sex	*n* (%)		*n* (%)	1.0000
Female	6,281 (61.2)	6,281 (61.2)	6,281 (61.2)	
Male	3,991 (38.8)	3,991 (38.8)	3,991 (38.8)	
Age, years				1.0000
18–29	1,553 (15.1)	1,553 (15.1)	1,553 (15.1)	
30–39	2,161 (21.0)	2,161 (21.0)	2,161 (21.0)	
40–49	1,538 (15.0)	1,538 (15.0)	1,538 (15.0)	
50–59	1,796 (17.5)	1,796 (17.5)	1796 (17.5)	
60–69	1,468 (14.3)	1,468 (14.3)	1,468 (14.3)	
70–79	1,248 (12.2)	1,248 (12.2)	1,248 (12.2)	
≥80	508 (5.0)	508 (5.0)	508 (5.0)	
Low income	139 (1.3)	139 (1.3)	139 (1.3)	1.0000
Types of surgery				1.0000
Skin	107 (1.0)	107 (1.0)	107 (1.0)	
Breast	142 (1.4)	142 (1.4)	142 (1.4)	
Musculoskeletal	2,427 (23.6)	2,427 (23.6)	2,427 (23.6)	
Respiratory	524 (5.1)	524 (5.1)	524 (5.1)	
Cardiovascular	172 (1.7)	172 (1.7)	172 (1.7)	
Digestive	2,161 (21.0)	2,161 (21.0)	2,161 (21.0)	
Kidney, ureter, bladder	718 (7.0)	718 (7.0)	718 (7.0)	
Delivery, CS, abortion	1,686 (16.4)	1,686 (16.4)	1,686 (16.4)	
Neurosurgery	865 (8.4)	865 (8.4)	865 (8.4)	
Eye	71 (0.7)	71 (0.7)	71 (0.7)	
Others	1,399 (13.6)	1,399 (13.6)	1,399 (13.6)	
Types of anesthesia				1.0000
General	6,809 (66.3)	6,809 (66.3)	6,809 (66.3)	
Regional	3,463 (33.7)	3,463 (33.7)	3,463 (33.7)	
Medical conditions				
Hypertension	2,052 (20.0)	2052 (20.0)	2052 (20.0)	1.0000
Mental disorders	1,449 (14.1)	1,449 (14.1)	1,449 (14.1)	1.0000
Diabetes	941 (9.2)	941 (9.2)	941 (9.2)	1.0000
Ischemic heart disease	463 (4.5)	463 (4.5)	463 (4.5)	1.0000
COPD	370 (3.6)	370 (3.6)	370 (3.6)	1.0000
Hyperlipidemia	271 (2.6)	271 (2.6)	271 (2.6)	1.0000
Liver cirrhosis	100 (1.0)	100 (1.0)	100 (1.0)	1.0000
Heart failure	92 (0.9)	92 (0.9)	92 (0.9)	1.0000
Renal dialysis	37 (0.4)	37 (0.4)	37 (0.4)	1.0000
Level of surgical risk				1.0000
Low risk	5,620 (54.7)	5,620 (54.7)	5,620 (54.7)	
Medium risk	4,160 (40.5)	4,160 (40.5)	4,160 (40.5)	
High risk	138 (1.3)	138 (1.3)	138 (1.3)	
Heart surgery	77 (0.8)	77 (0.8)	77 (0.8)	
Other	277 (2.7)	277 (2.7)	277 (2.7)	

COPD, chronic obstructive pulmonary disease; CS, cesarean section.

In the logistic regression model (Table 2), surgical patients with a recent history of influenza within 1–14 days (OR 1.27, 95% CI 0.87–1.86) or 15–30 days (OR 0.84, 95% CI 0.55–1.28) had no increased risk of postoperative mortality when compared with surgical patients without influenza. However, surgical patients with a recent history of influenza within 1–14 days had an increased risk of pneumonia (OR 1.74, 95% CI 1.45–2.09), septicemia (OR 1.54, 95% CI 1.34–1.76), pulmonary embolism (OR 8.99, 95% CI 1.14–71.2), acute renal failure (OR 1.68, 95% CI 1.22–2.31), or urinary tract infection (OR 1.28, 95% CI 1.11–1.46) compared with those who had no influenza. An increased risk of admission to intensive care (OR 1.59, 95% CI 1.43–1.77), prolonged length of stay (8.2 ± 10.4 vs. 7.1 ± 8.7, *p* < 0.0001), and higher medical expenditures ($2,794 ± 4,039 vs. $2,496 ± 3,574 USD, *p* < 0.0001) were also noted in patients with a recent history of influenza within 1–14 days as compared to those without influenza.

**Table 2:** Adverse outcomes of surgical patients with and without influenza.

	No influenza (*N* = 10,272)			Influenza 15–30 days (*N* = 10,272)			Influenza 1–14 days (*N* = 10,272)		
	Event	%	OR (95% CI)^a^	Event	%	OR (95% CI)^a^	Event	%	OR (95% CI)^a^
30-day in-hospital mortality	48	0.5	1.00 (reference)	42	0.4	0.84 (0.55–1.28)	62	0.6	1.27 (0.87–1.86)
Postoperative complications									
Pneumonia	195	1.9	1.00 (reference)	207	2.0	1.02 (0.84–1.26)	333	3.2	1.74 (1.45–2.09)
Septicemia	369	3.6	1.00 (reference)	424	4.0	1.12 (0.97–1.29)	564	5.4	1.54 (1.34–1.76)
Pulmonary embolism	1	0.01	1.00 (reference)	1	0.01	0.98 (0.06–15.7)	9	0.1	8.99 (1.14–71.2)
Acute renal failure	63	0.6	1.00 (reference)	49	0.5	0.74 (0.50–1.08)	106	1.0	1.68 (1.22–2.31)
Stroke	227	2.2	1.00 (reference)	175	1.7	0.72 (0.81–1.19)	231	2.2	0.98 (0.81–1.19)
Urinary tract infection	424	4.1	1.00 (reference)	473	4.5	1.09(0.95–1.26)	542	5.2	1.28 (1.11–1.46)
Deep wound infection	46	0.5	1.00 (reference)	48	0.5	1.01 (0.68–1.52)	50	0.5	1.06 (0.71–1.58)
AMI	21	0.2	1.00 (reference)	28	0.3	1.32 (0.73–2.42)	30	0.3	1.47 (0.81–2.66)
Postoperative bleeding	51	0.5	1.00 (reference)	57	0.5	1.09 (0.75–1.59)	56	0.5	1.07 (0.73–1.57)
ICU stay	898	8.7	1.00 (reference)	988	9.4	1.09 (0.98–1.22)	1,252	11.9	1.59 (1.43–1.77)
ME, USD^‡^	2,496 ± 3,574			2,540 ± 3,583			2,794 ± 4,039		
LOS, days^‡^	7.1 ± 8.7			7.4 ± 9.4			8.2 ± 10.4		

AMI, Acute myocardial infarction; CI, confidence interval; LOS, Length of hospital stay; ME, Medical expenditure; OR, odds ratio. ^a^Calculated by logistic regression. ^‡^Mean ± SD; Linear regressions were used to evaluate the medical expenditure (beta = 144.50306, *p* < 0.0001) and length of hospital stay (beta = 0.53323, *p* < 0.0001) associated with influenza.

**Figure 1:**
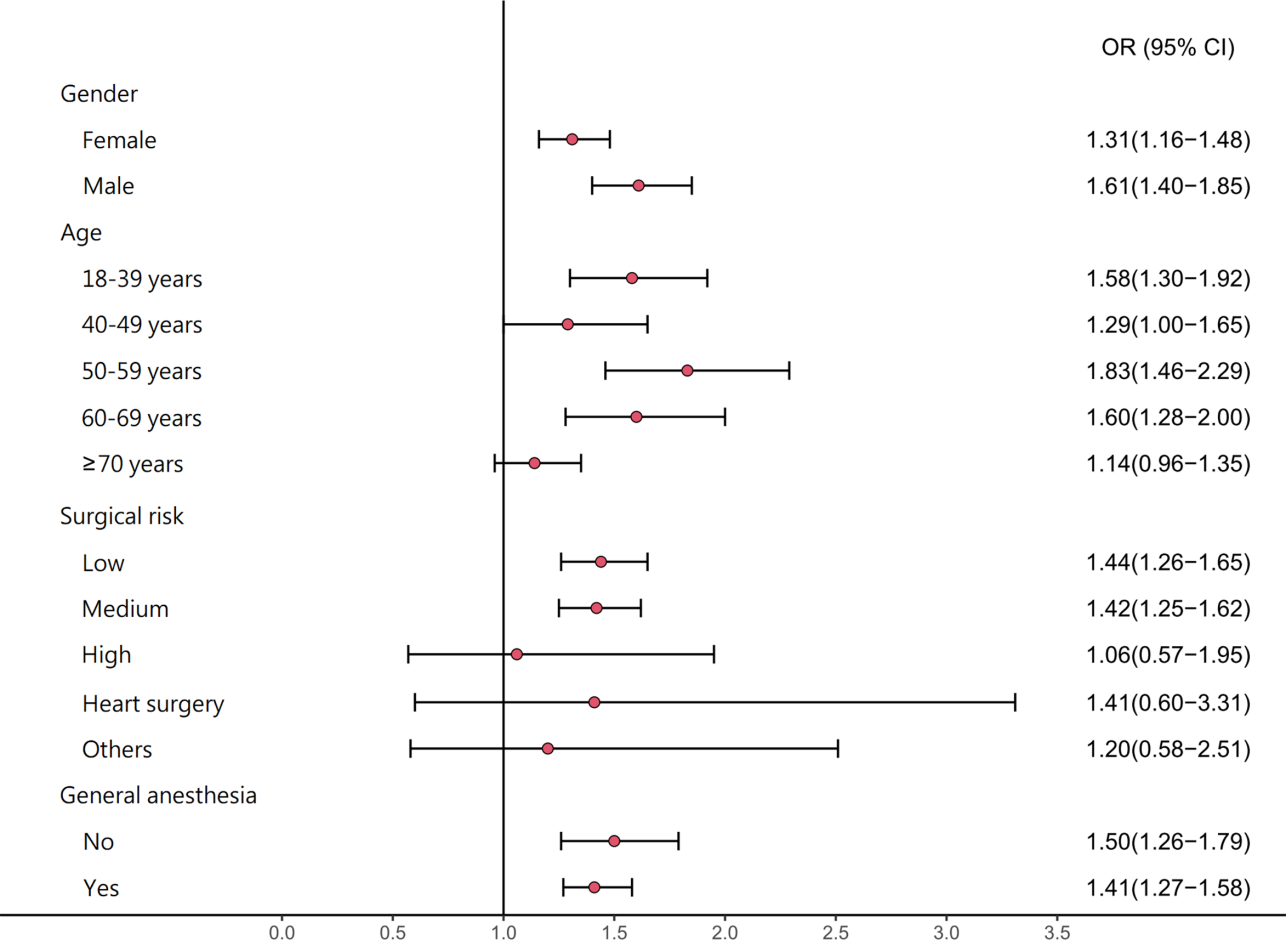
The stratified analysis for the adjusted odds ratios (95% confidence intervals) of postoperative adverse events associated with influenza.

As shown in Figure 1, a recent history of influenza infection within 1–14 days was associated with postoperative adverse events in women (OR 1.31, 95% CI 1.16–1.48), men (OR 1.61, 95% CI 1.40–1.85), and people in the age groups 18–39 years (OR 1.58, 95% CI 1.30–1.92), 40–49 years (OR 1.29, 95% CI 1.00–1.65), 50–59 years (OR 1.83, 95% CI 1.46–2.29), and 60–69 years (OR 1.60, 95% CI 1.28–2.00). The association between a recent history of influenza within 1–14 days and postoperative adverse events was significant in patients who received general anesthesia (OR 1.41, 95% CI 1.27–1.58), regional anesthesia (OR 1.50, 95% CI 1.26–1.79), and those undergoing low-risk (OR 1.44, 95% CI 1.26–1.65) or medium-risk (OR 1.42, 95% CI 1.25–1.62) surgery. Additional information for stratified analysis for the association between influenza and postoperative adverse events could be achieved in Supplementary Table S2.

**Figure 2:**
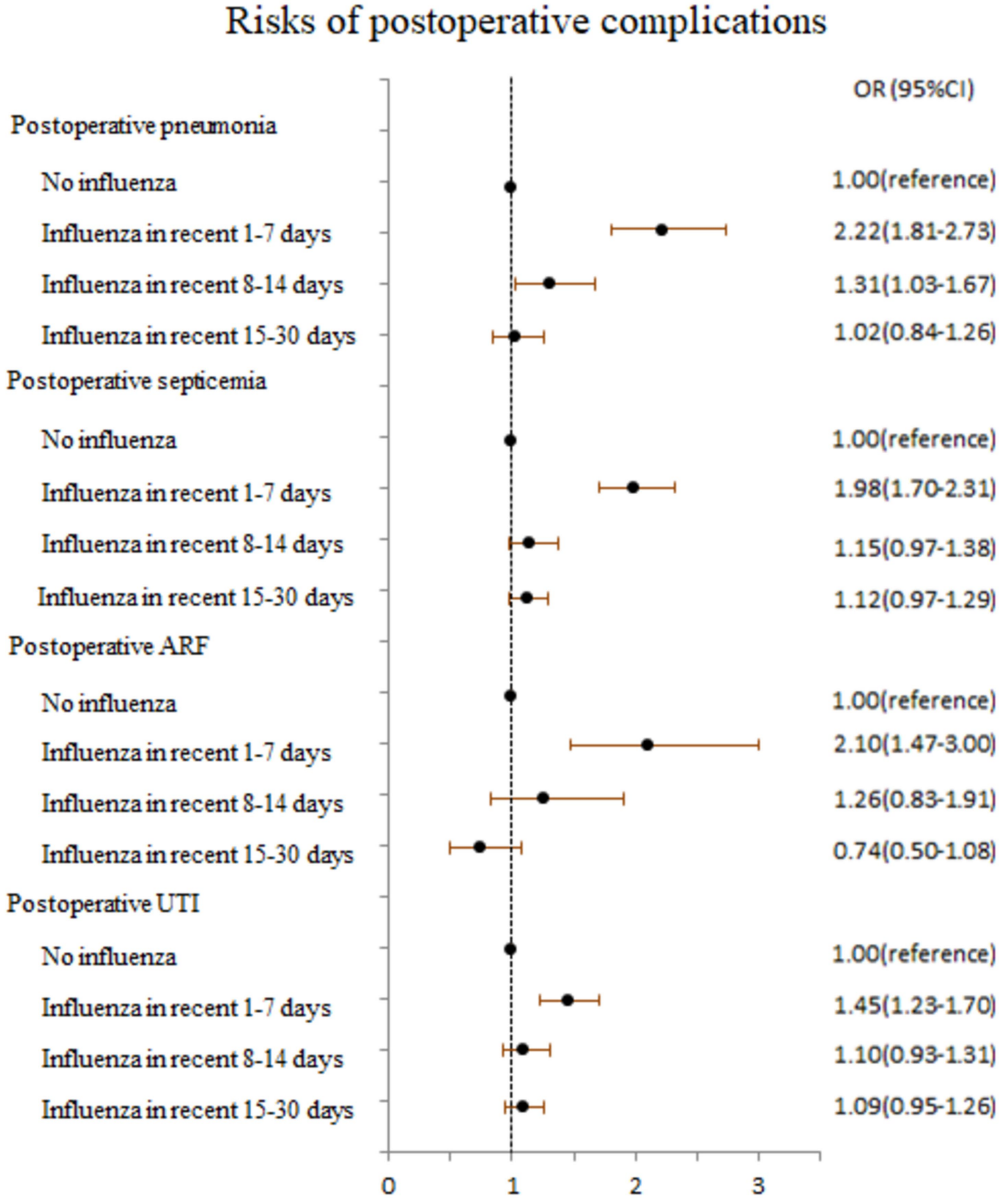
Risks of postoperative pneumonia, septicemia, acute renal failure (ARF), and urinary tract infection (UTI) in association with time period of recent influenza.

Preoperative influenza occurring within 1–7 days (OR 1.78, 95% CI 1.60–1.98) and 8–14 days (OR 1.14, 95% CI 1.02–1.28) was associated with adverse events after surgery (Table 3). We found the increased risk of postoperative adverse events in patients with influenza within 1–14 days preoperatively who had or had no influenza vaccination compared with those had no influenza. As shown in Figure 2, the adjusted ORs for a recent history of influenza within 1–7 days preoperatively for postoperative pneumonia, septicemia, acute renal failure, or urinary tract infection were 2.22 (95% CI 1.81–2.73), 1.98 (95% CI 1.70–2.31), 2.10 (95% CI 1.47–3.00), and 1.45 (95% CI 1.23–1.70), respectively.

**Table 3:** Adverse events in association with the characteristics of influenza.

	Adverse events^*^			
	*n*	Events	Incidence, %	OR (95% CI)^†^
No influenza	10,272	925	9.0	1.00 (reference)
Preoperative of influenza				
Influenza in recent 1–7 days	5,071	775	15.3	1.78 (1.60–1.98)
Influenza in recent 8–14 days	5,201	530	10.2	1.14 (1.02–1.28)
Influenza in recent 15–30 days	10,272	1,017	9.9	1.08 (0.98–1.19)
Patients with influenza 1–14 days				
Had no vaccination	8,854	1,040	11.8	1.49 (1.35–1.64)
Received vaccination‡	1,418	161	11.4	1.29 (1.06–1.57)

CI, confidence interval; IV, Influenza vaccine; OR, odds ratio. ^*^Adverse events included with pneumonia, septicemia, acute renal failure, urinary tract infection. ^†^Adjusted for all covariates listed in Table 1. ^‡^Within previous one year before surgery.

## Discussion

In this propensity-score matched study based on a real-world database, we found that patients with influenza within 14 days prior to surgery, and particularly those with influenza within 7 days prior to surgery, had increased risks of postoperative pneumonia, septicemia, acute renal failure, and urinary tract infection compared with those who did not. Prolonged length of hospital stays, increased medical expenditures, and increased chances of admission to intensive care following surgery were also found among patients with influenza as compared to those in the non-influenza control group. The association between influenza and postoperative adverse events was also found in varying subgroups, such as both sexes, age groups, those with comorbid underlying medical conditions, surgical risk, and anesthesia method.

Influenza viruses can infect not only the upper respiratory epithelium but also the lower respiratory tract (21). It can replicate in the tracheobronchial epithelium and cause viral pneumonia (21), which is the most common complication caused by influenza. In nasopharyngeal epithelial cells, the presence of influenza viruses is associated with increased *S. pneumoniae* colonization as well as increased bacterial attachment sites, possibly leading to secondary pneumococcal pneumonia (22). Besides the viral pneumonia caused by influenza viruses, damage to the respiratory tract may predispose individuals to secondary bacterial pneumonia and subsequent mortality.

As a rule, we would recommend that patients who have had influenza prior to surgery postpone surgery for 1 to 2 weeks. In pediatric surgical patients, preoperative respiratory history (nocturnal dry cough or wheezing ≥3 times in 12 months before surgery) and upper airway infection increase adverse perioperative respiratory events in children (12). To the best of our knowledge, there is minimal prior evidence or data on complications after surgery among adult patients who have had influenza prior to surgery. This study demonstrated that having had influenza prior to surgery increases the risk of postoperative pneumonia. This influence was significant within the first two weeks of infection.

According to previous studies, some patients have a higher risk of complications after influenza. These patients had the following characteristics: old age (over 65 years), pregnant or being within 2 weeks after delivery, residing in long-term care facilities, morbid obesity, chronic medical conditions (e.g., immunocompromising conditions, chronic lung disease, chronic heart disease, or chronic kidney disease), or receiving glucocorticoids or other immunosuppressive medications (23–27). Distinct from the patients mentioned above, our study revealed that influenza also had an influence on most of the surgical patients.

After influenza, the immune response shows similar pathways as with bacterial infection (28). Damage from the immune system response (21) and impaired antibacterial defenses (such as neutrophil apoptosis, inhibition of chemotaxis, and suppression of phagocytosis) (29) can lead to endothelial damage and microvascular permeability changes (30). Such responses are similar to those in bacterial infection that may further cause tissue edema and organ failure. A retrospective study estimated that influenza-associated sepsis in the USA is about 8.8 per 100,000 person-years (95% CI 3.9–16.5). Severe sepsis occurred in 73% of patients with influenza-associated critical illness, including acute respiratory failure, severe sepsis, or in-hospital death (31). A large prospective, multinational, and multicenter cross-sectional study in Southeast Asia found that 4% of all sepsis cases (*n* = 1,578) were due to influenza infection (32). In this study, the risk of postoperative septicemia was significant. Our study result was in agreement with previous studies referenced previously.

Post-influenza acute renal injury or failure was unusual. Previous reports were mostly case reports or small case series (33, 34). Pandemic influenza A (H1N1) 2009 virus infection, similar to seasonal influenza, worsened underlying medical conditions, including in the respiratory, cardiac, neurologic, and musculoskeletal systems, and increased the risk of secondary bacterial infections (35, 36). Different from seasonal influenza, influenza A (H1N1) 2009 has been shown to increase the risk of acute renal injury in critically-ill patients. The actual pathophysiologic mechanisms for the acute renal injury in patients with influenza are not known. Some theories for this have been suggested, including acute tubular necrosis contributing to renal hypoperfusion; rhabdomyolysis-related renal injury; or direct viral injury to the kidney (37–39). In patients hospitalized for influenza A (H1N1) 2009, the incidence of acute kidney injury was about 30–40% according to previous reports (40, 41). In our study, we demonstrated an increased risk of acute renal failure in surgical patients with influenza; the risk increased when the infection occurred within 7 days prior to surgery.

Limited information was available on postoperative mortality in patients who had recent influenza. The respiratory infection (respiratory symptoms, fever, and with antibiotics treatment) within one month before surgery was associated with increased risk of postoperative pulmonary complications (including postoperative respiratory infection, respiratory failure, pleural effusion, atelectasis, pneumothorax, bronchospasm, or aspiration pneumonitis). The thirty-day mortality was higher in patients with a complication when compared to those without influenza (15). However, our study revealed that the risk of postoperative 30-day in-hospital mortality was neither increased in the group of influenza 1–14 days nor the group of influenza 15–30 days when compared with patients without influenza. Our result being different from the previous study might be due to the different pre-surgical respiratory infection conditions (1–14 days and 15–30 days).

In Taiwan’s current medical practice guidelines and recommendations, there are no suggestions or statements for patients with influenza infection who receive surgery. Clinical physicians need to report to Taiwan’s Centers for Disease Control when their patients have influenza with severe complications. In the clinical settings, surgeons and anesthesiologists may discuss the possibility of postponing surgery for patients infected with influenza considering case-by-case factors, including the patient’s condition, the type of surgery, and the risk of complications. If a patient has a mild case of influenza, surgery may not be postponed in the usual clinical settings in Taiwan. The findings of our study provide the scientific evidence to show the potential risk of postoperative complications among patients with influenza who receive surgery. We suggest that surgery might need to be postponed or the patient’s condition fully considered for patients who had influenza infection before surgery.

Some study limitations must be noted with regard to interpreting our findings. First, the insurance database lacked information on sociodemographic characteristics, lifestyle information (e.g., smoking, alcohol drinking, and physical activity), laboratory data, and detailed characteristics of surgery (such as operation time, blood loss, and transfusion amount). Therefore, we were not able to evaluate their potential impact on perioperative outcomes in this study. Second, misclassification might have occurred in our study, because we were not able to definitively demonstrate that the non-influenza control group had not been infected with influenza. Some influenza-infected people may have been categorized as controls, if they lacked typical influenza symptoms and may have instead had been labeled as having the ‘common cold’. Thus, the impact of influenza on perioperative outcomes may have been underestimated in this study. Third, the severity and type of influenza may have varying influence on perioperative outcomes, but we were not able to evaluate either the severity or type of influenza in this study. Fourth, the current data in this study is a decade old, which could not present the latest status of the clinical situation in Taiwan. Fifth, we could not exclude the possibility that there may be false positive cases existing in the case group of influenza in this study. In addition, patients with severe influenza may not end up undergoing surgery after evaluation by the surgical team. Thus, the influenza group in this study may include patients who have minor signs or symptoms of influenza. Finally, the use of the propensity-matching method had its own limitations, especially regarding randomization and intended bias observed. Residual confounding effects are also possible in this study. Thus, caution was needed in interpreting the findings of this study.

In conclusion, we found that patients with a history of influenza within 14 days prior to surgery had increased risk of postoperative complications and increased medical resources utilization, particularly those patients who had influenza within 7 days prior to surgery. Our findings suggest that the surgical care team should consider the risks and benefits for patients with influenza who are planned to undergo major surgery.

## Data availability statement

The datasets presented in this article are not readily available because the data that support the findings of this study are available from the Ministry of Health and Welfare, Taiwan but restrictions apply to the availability of these data, which were used under license for the current study, and so are not publicly available. Data are however available from the authors upon reasonable request and with permission of the Ministry of Health and Welfare, Taiwan. Researchers could contact with the liaison and make application for the database by the following information: https://dep.mohw.gov.tw/dos/cp-2516-59203-113.html. Requests to access the datasets should be directed to The Ministry of Health and Welfare, Taiwan.

## Ethics statement

The studies involving human participants were reviewed and approved by the institutional review board of Taipei Medical University (TMU-JIRB-202302005; TMU-JIRB-201910032; TMU-JIRB-201905042; TMU-JIRB-201902053) According to the regulations of the Ministry of Health and Welfare, the electronic database was decoded and patient identifiers scrambled for further research access; therefore, the personal privacy of patients was protected and informed consent was not required. Written informed consent for participation was not required for this study in accordance with the national legislation and the institutional requirements.

## Author contributions

FL, C-CL, T-LC, and H-YC conceived the study. FL, C-CL, and H-YC completed the study design, data acquisition, and statistical analysis. FL, C-CL, T-LC, Y-MH, Y-JL, and H-YC contributed to the study design, interpretation of data, reviewed, and contributed to the revision of the manuscript for important intellectual content. FL and C-CL drafted the manuscript. H-YC had full access to all of the data in the study and takes responsibility for the integrity of the data and the accuracy of the data analysis. All authors read and approved the final manuscript.

## Funding

This study was supported in part by the Ministry of Science and Technology, Taiwan (MOST111-2320-B-532 -001-MY3; MOST110-2314-B-038-108-MY2; MOST108-2320-B-038-070-MY3).

## Conflict of interest

The authors declare that the research was conducted in the absence of any commercial or financial relationships that could be construed as a potential conflict of interest.

## Publisher’s note

All claims expressed in this article are solely those of the authors and do not necessarily represent those of their affiliated organizations, or those of the publisher, the editors and the reviewers. Any product that may be evaluated in this article, or claim that may be made by its manufacturer, is not guaranteed or endorsed by the publisher.

## References

[1] Iuliano AD Roguski KM Chang HH Muscatello DJ Palekar R Tempia S. Estimates of global seasonal influenza-associated respiratory mortality: a modelling study. Lancet. (2018) 391:1285–300. doi: 10.1016/S0140-6736(17)33293-2, PMID: 29248255 PMC5935243

[2] World Health Organization. (2018). Influenza (seasonal) fact sheet. Available at: http://www.who.int/mediacentre/factsheets/fs211/en/ (Accessed November 6, 2018)

[3] Molinari NA Ortega-Sanchez IR Messonnier ML Thompson WW Wortley PM Weintraub E. The annual impact of seasonal influenza in the US: measuring disease burden and costs. Vaccine. (2007) 25:5086–96. doi: 10.1016/j.vaccine.2007.03.046, PMID: 17544181

[4] Fan VY Jamison DT Summers LH. Pandemic risk: how large are the expected losses? Bull World Health Organ. (2018) 96:129–34. doi: 10.2471/BLT.17.199588, PMID: 29403116 PMC5791779

[5] Panhwar MS Kalra A Gupta T Kolte D Khera S Bhatt DL. Effect of Influenza on outcomes in patients with heart failure. JACC Heart Fail. (2019) 7:112–7. doi: 10.1016/j.jchf.2018.10.011 30611718

[6] Mulpuru S Li L Ye L Hatchette T Andrew MK Ambrose A. Effectiveness of Influenza vaccination on hospitalizations and risk factors for severe outcomes in hospitalized patients with COPD. Chest. (2019) 155:69–78. doi: 10.1016/j.chest.2018.10.044, PMID: 30616737

[7] Bowman BT Rosner MH. Influenza and the patient with end-stage renal disease. J Nephrol. (2018) 31:225–30. doi: 10.1007/s40620-017-0407-9, PMID: 28528400

[8] Toschke AM Heuschmann PU Wood O Wolfe CD. Temporal relationship between influenza infections and subsequent first-ever stroke incidence. Age Ageing. (2009) 38:100–3. doi: 10.1093/ageing/afn232, PMID: 19029068

[9] Smeeth L Thomas SL Hall AJ Hubbard R Farrington P Vallance P. Risk of myocardial infarction and stroke after acute infection or vaccination. N Engl J Med. (2004) 351:2611–8. doi: 10.1056/NEJMoa041747 15602021

[10] Lee KR Bae JH Hwang IC Kim KK Suh HS Ko KD. Effect of Influenza vaccination on risk of stroke: a systematic review and Meta-analysis. Neuroepidemiology. (2017) 48:103–10. doi: 10.1159/000478017, PMID: 28628919

[11] Weiser TG Haynes AB Molina G Lipsitz SR Esquivel MM Uribe-Leitz T. Size and distribution of the global volume of surgery in 2012. Bull World Health Organ. (2016) 94:201–209F. doi: 10.2471/BLT.15.159293, PMID: 26966331 PMC4773932

[12] Malviya S Voepel-Lewis T Siewert M Pandit UA Riegger LQ Tait AR. Risk factors for adverse postoperative outcomes in children presenting for cardiac surgery with upper respiratory tract infections. Anesthesiology. (2003) 98:628–32. doi: 10.1097/00000542-200303000-00009, PMID: 12606905

[13] von Ungern-Sternberg BS Boda K Chambers NA Rebmann C Johnson C Sly PD. Risk assessment for respiratory complications in paediatric anaesthesia: a prospective cohort study. Lancet. (2010) 376:773–83. doi: 10.1016/S0140-6736(10)61193-2 20816545

[14] Canet J Sanchis J Briones Z Paluzié G Sabaté S. Recent acute respiratory tract infection in adults is a significant risk factor of postoperative complications. Eur J Anaesthesiol. (2008) 25:72–3. doi: 10.1097/00003643-200805001-0022918228639

[15] Canet J Gallart L Gomar C Paluzie G Vallès J Castillo J. Prediction of postoperative pulmonary complications in a population-based surgical cohort. Anesthesiology. (2010) 113:1338–50. doi: 10.1097/ALN.0b013e3181fc6e0a 21045639

[16] Liao CC Shen WW Chang CC Chang H Chen TL. Surgical adverse outcomes in patients with schizophrenia: a population-based study. Ann Surg. (2013) 257:433–8. doi: 10.1097/SLA.0b013e31827b9b25 23241870

[17] Yeh CC Liao CC Chang YC Jeng LB Yang HR Shih CC. Adverse outcomes after noncardiac surgery in patients with diabetes: a nationwide population-based retrospective cohort study. Diabetes Care. (2013) 36:3216–21. doi: 10.2337/dc13-0770, PMID: 23990518 PMC3781492

[18] Hsing AW Ioannidis JP. Nationwide population science: lessons from the Taiwan National Health Insurance Research Database. JAMA Intern Med. (2015) 175:1527–9. doi: 10.1001/jamainternmed.2015.3540 26192815

[19] Kristensen SD Knuuti J Saraste A Anker S Bøtker HE De Hert S. 2014 ESC/ESA guidelines on non-cardiac surgery: cardiovascular assessment and management: the joint task force on non-cardiac surgery: cardiovascular assessment and management of the European Society of Cardiology (ESC) and the European Society of Anaesthesiology (ESA). Eur J Anaesthesiol. (2014) 31:517–73. doi: 10.1097/EJA.0000000000000150 25127426

[20] Roques F Nashef SA Michel P Gauducheau E de Vincentiis C Baudet E. Risk factors and outcome in European cardiac surgery: analysis of the EuroSCORE multinational database of 19030 patients. Eur J Cardiothorac Surg. (1999) 15:816; discussion 822-3.–23. doi: 10.1016/s1010-7940(99)00106-2, PMID: 10431864

[21] Kalil AC Thomas PG. Influenza virus-related critical illness: pathophysiology and epidemiology. Crit Care. (2019) 23:258. doi: 10.1186/s13054-019-2539-x, PMID: 31324202 PMC6642581

[22] Wolter N Tempia S Cohen C Madhi SA Venter M Moyes J. High nasopharyngeal pneumococcal density, increased by viral coinfection, is associated with invasive pneumococcal pneumonia. J Infect Dis. (2014) 210:1649–57. doi: 10.1093/infdis/jiu326, PMID: 24907383

[23] Jain S Kamimoto L Bramley AM Schmitz AM Benoit SR Louie J. Hospitalized patients with 2009 H1N1 influenza in the United States, April-June 2009. N Engl J Med. (2009) 361:1935–44. doi: 10.1056/NEJMoa0906695, PMID: 19815859

[24] Chaves SS Aragon D Bennett N Cooper T D'Mello T Farley M. Patients hospitalized with laboratory-confirmed influenza during the 2010-2011 influenza season: exploring disease severity by virus type and subtype. J Infect Dis. (2013) 208:1305–14. doi: 10.1093/infdis/jit316, PMID: 23863950

[25] Kwong JC Campitelli MA Rosella LC. Obesity and respiratory hospitalizations during influenza seasons in Ontario, Canada: a cohort study. Clin Infect Dis. (2011) 53:413–21. doi: 10.1093/cid/cir442, PMID: 21844024 PMC3156143

[26] Influenza Investigators ANZIC Webb SA Pettilä V Seppelt I Bellomo R Bailey M. Critical care services and 2009 H1N1 influenza in Australia and New Zealand. N Engl J Med. (2009) 361:1925–34. doi: 10.1056/NEJMoa0908481 19815860

[27] Walker TA Waite B Thompson MG McArthur C Wong C Baker MG. Risk of severe Influenza among adults with chronic medical conditions. J Infect Dis. (2020) 221:183–90. doi: 10.1093/infdis/jiz570 31678990

[28] Teijaro JR Walsh KB Cahalan S Fremgen DM Roberts E Scott F. Endothelial cells are central orchestrators of cytokine amplification during influenza virus infection. Cells. (2011) 146:980–91. doi: 10.1016/j.cell.2011.08.015, PMID: 21925319 PMC3176439

[29] Florescu DF Kalil AC. The complex link between influenza and severe sepsis. Virulence. (2014) 5:137–42. doi: 10.4161/viru.27103, PMID: 24253109 PMC3916367

[30] Steinberg BE Goldenberg NM Lee WL. Do viral infections mimic bacterial sepsis? The role of microvascular permeability: a review of mechanisms and methods. Antivir Res. (2012) 93:2–15. doi: 10.1016/j.antiviral.2011.10.019, PMID: 22068147

[31] Ortiz JR Neuzil KM Shay DK Rue TC Neradilek MB Zhou H. The burden of influenza-associated critical illness hospitalizations. Crit Care Med. (2014) 42:2325–32. doi: 10.1097/CCM.0000000000000545, PMID: 25148596 PMC4620028

[32] Southeast Asia Infectious Disease Clinical Research Network. Causes and outcomes of sepsis in Southeast Asia: a multinational multicentre cross-sectional study. Lancet Glob Health. (2017) 5:e157–67. doi: 10.1016/S2214-109X(17)30007-4, PMID: 28104185 PMC5332551

[33] Watanabe T Yoshikawa H Abe Y Yamazaki S Uehara Y Abe T. Renal involvement in children with influenza a virus infection. Pediatr Nephrol. (2003) 18:541–4. doi: 10.1007/s00467-003-1143-z, PMID: 12698331

[34] West SD Brunskill NJ. Complications associated with influenza infection. Postgrad Med J. (2002) 78:100–8. doi: 10.1136/pmj.78.916.100, PMID: 11807195 PMC1742281

[35] Rothberg MB Haessler SD. Complications of seasonal and pandemic influenza. Crit Care Med. (2010) 38:e91–7. doi: 10.1097/CCM.0b013e3181c92eeb 19935413

[36] Sullivan SJ Jacobson RM Dowdle WR Poland GA. 2009 H1N1 influenza. Mayo Clin Proc. (2010) 85:64–76. doi: 10.4065/mcp.2009.0588, PMID: 20007905 PMC2800287

[37] Sood MM Rigatto C Zarychanski R Komenda P Sood AR Bueti J. Acute kidney injury in critically ill patients infected with 2009 pandemic influenza a(H1N1): report from a Canadian Province. Am J Kidney Dis. (2010) 55:848–55. doi: 10.1053/j.ajkd.2010.01.011, PMID: 20303633 PMC7125797

[38] Trimarchi H Greloni G Campolo-Girard V Giannasi S Pomeranz V San-Roman E. H1N1 infection and the kidney in critically ill patients. J Nephrol. (2010) 23:725–31. doi: 10.1093/ndtplus/sfp142, PMID: 20349409

[39] Watanabe T. Renal complications of seasonal and pandemic influenza a virus infections. Eur J Pediatr. (2013) 172:15–22. doi: 10.1007/s00431-012-1854-x, PMID: 23064728

[40] Demirjian SG Raina R Bhimraj A Navaneethan SD Gordon SM Schreiber MJ Jr. Influenza a infection and acute kidney injury: incidence, risk factors, and complications. Am J Nephrol. (2009, 2011) 34:1–8. doi: 10.1159/000328386, PMID: 21625080

[41] Perez-Padilla R de la Rosa-Zamboni D Ponce de Leon S Hernandez M Quiñones-Falconi F Bautista E. Pneumonia and respiratory failure from swine-origin influenza a (H1N1) in Mexico. N Engl J Med. (2009) 361:680–9. doi: 10.1056/NEJMoa0904252, PMID: 19564631

