# Supplementary material for: Outcomes after surgery in patients with and without recent influenza: a nationwide population-based study
Source: Front Med (Lausanne). 2023 Jun 9;10:1117885. doi: 10.3389/fmed.2023.1117885 (PMC10288488; doi:10.3389/fmed.2023.1117885)
Supplement: Supplementary file 1 [file Data_Sheet_1.docx]

| **Table S1. Characteristics of surgical patients with and without influenza (before matching)** | | | | | | | |
| --- | --- | --- | --- | --- | --- | --- | --- |
|  | No influenza  (N=3154730) | | Influenza 15-30 days  (N=13992) | | Influenza 1-14 days  (N=14147) | | p-value |
| Sex | n | (%) | n | (%) | n | (%) | <0.0001 |
| Female | 1695921 | (53.8) | 8199 | (58.6) | 8177 | (57.8) |  |
| Male | 1458809 | (46.2) | 5793 | (41.4) | 5970 | (42.2) |  |
| Age, years |  |  |  |  |  |  | <0.0001 |
| 18-29 | 409418 | (13.0) | 1809 | (12.9) | 1893 | (13.4) |  |
| 30-39 | 560908 | (17.8) | 2593 | (18.5) | 2458 | (17.4) |  |
| 40-49 | 527583 | (16.7) | 2049 | (14.6) | 2121 | (15.0) |  |
| 50-59 | 579211 | (18.4) | 2537 | (18.1) | 2565 | (18.1) |  |
| 60-69 | 462879 | (14.7) | 2252 | (16.1) | 2100 | (14.8) |  |
| 70-79 | 396935 | (12.6) | 1905 | (13.6) | 2025 | (14.3) |  |
| ≥80 | 217796 | (6.9) | 847 | (6.1) | 985 | (7.0) |  |
| Low income | 73157 | (2.3) | 357 | (2.6) | 386 | (2.7) | 0.0011 |
| Types of surgery |  |  |  |  |  |  | <0.0001 |
| Skin | 51605 | (1.6) | 197 | (1.4) | 193 | (1.4) |  |
| Breast | 55055 | (1.8) | 217 | (1.6) | 179 | (1.3) |  |
| Musculoskeletal | 887506 | (28.1) | 3376 | (24.1) | 3284 | (23.2) |  |
| Respiratory | 140643 | (4.5) | 793 | (5.7) | 790 | (5.6) |  |
| Cardiovascular | 81493 | (2.6) | 356 | (2.5) | 497 | (3.5) |  |
| Digestive | 630704 | (20.0) | 2821 | (20.2) | 3235 | (22.9) |  |
| Kidney, ureter, bladder | 226664 | (7.2) | 1102 | (7.9) | 1069 | (7.6) |  |
| Delivery, CS, abortion | 321965 | (10.2) | 1827 | (13.1) | 1805 | (12.8) |  |
| Neurosurgery | 287040 | (9.1) | 1228 | (8.8) | 1201 | (8.5) |  |
| Eye | 36175 | (1.2) | 140 | (1.0) | 118 | (0.8) |  |
| Others | 435880 | (13.8) | 1935 | (13.8) | 1776 | (12.6) |  |
| Types of anesthesia |  |  |  |  |  |  | 0.0038 |
| General | 2183705 | (69.2) | 9543 | (68.2) | 9677 | (68.4) |  |
| Regional | 971025 | (30.8) | 4449 | (31.8) | 4470 | (31.6) |  |
| Medical conditions |  |  |  |  |  |  |  |
| Hypertension | 649264 | (20.6) | 3389 | (24.2) | 3418 | (24.2) | <0.0001 |
| Diabetes | 359311 | (11.4) | 1818 | (13.0) | 1898 | (13.4) | <0.0001 |
| Hyperlipidemia | 134520 | (4.3) | 736 | (5.3) | 690 | (4.9) | <0.0001 |
| Mental disorders | 460508 | (14.6) | 2676 | (19.1) | 2491 | (17.6) | <0.0001 |
| Ischemic heart disease | 211070 | (6.7) | 1145 | (8.2) | 1109 | (7.8) | <0.0001 |
| Heart failure | 58247 | (1.9) | 330 | (2.4) | 338 | (2.4) | <0.0001 |
| COPD | 130191 | (4.1) | 922 | (6.6) | 856 | (6.1) | <0.0001 |
| Liver cirrhosis | 67437 | (2.1) | 324 | (2.3) | 357 | (2.5) | 0.0024 |
| Renal dialysis | 50545 | (1.6) | 156 | (1.1) | 152 | (1.1) | <0.0001 |
| Level of surgical risk |  |  |  |  |  |  | <0.0001 |
| Low risk | 1701438 | (53.9) | 7553 | (54.0) | 7266 | (51.4) |  |
| Medium risk | 1272182 | (40.3) | 5573 | (39.8) | 5995 | (42.4) |  |
| High risk | 55906 | (1.8) | 272 | (1.9) | 286 | (2.0) |  |
| Heart surgery | 24706 | (0.8) | 149 | (1.1) | 199 | (1.4) |  |
| Others | 100498 | (3.2) | 445 | (3.2) | 401 | (2.8) |  |
|  | | | | | | | |

**Figure S1. Selection of Study Patients**

3,182,869 surgical patients aged ≥ 18 years who underwent major surgery in 2008-2013

14147 patients had influenza within preoperative 1-14 days

3154730 patients without influenza preoperatively

Propensity-score matching procedure with case-control ratio=1: 1: 1

Evaluating postoperative complications and mortality

13992 patients had influenza within preoperative 15-30 days

10272 patients had influenza within preoperative 1-14 days

10272 patients without influenza preoperatively

10272 patients had influenza within preoperative 15-30 days

| **Table S2. Stratified analysis for the association between influenza and postoperative adverse events** | | | | | | |
| --- | --- | --- | --- | --- | --- | --- |
|  | | Adverse events* | | | | |
|  |  | n | Events | Incidence, % | OR | (95% CI)† |
| Female | No influenza | 6281 | 515 | 8.2 | 1.00 | (reference) |
|  | Influenza 1-14 days | 6281 | 664 | 10.3 | 1.31 | (1.16-1.48) |
| Male | No influenza | 3991 | 410 | 10.3 | 1.00 | (reference) |
|  | Influenza 1-14 days | 3991 | 624 | 15.2 | 1.61 | (1.40-1.85) |
| Age 18-39 years | No influenza | 3714 | 182 | 4.9 | 1.00 | (reference) |
|  | Influenza 1-14 days | 3714 | 280 | 7.4 | 1.58 | (1.30-1.92) |
| Age 40-49 years | No influenza | 1538 | 127 | 8.3 | 1.00 | (reference) |
|  | Influenza 1-14 days | 1538 | 162 | 10.3 | 1.29 | (1.00-1.65) |
| Age 50-59 years | No influenza | 1796 | 140 | 7.8 | 1.00 | (reference) |
|  | Influenza 1-14 days | 1796 | 243 | 13.2 | 1.83 | (1.46-2.29) |
| Age 60-69 years | No influenza | 1468 | 152 | 10.4 | 1.00 | (reference) |
|  | Influenza 1-14 days | 1468 | 230 | 15.2 | 1.60 | (1.28-2.00) |
| Age ≥70 years | No influenza | 1756 | 324 | 18.5 | 1.00 | (reference) |
|  | Influenza 1-14 days | 1756 | 373 | 20.4 | 1.14 | (0.96-1.35) |
| Low surgical risk | No influenza | 5620 | 397 | 7.1 | 1.00 | (reference) |
|  | Influenza 1-14 days | 5620 | 567 | 9.9 | 1.44 | (1.26-1.65) |
| Medium surgical risk | No influenza | 4160 | 475 | 11.4 | 1.00 | (reference) |
|  | Influenza 1-14 days | 4160 | 659 | 15.4 | 1.42 | (1.25-1.62) |
| High surgical risk | No influenza | 138 | 26 | 18.8 | 1.00 | (reference) |
|  | Influenza 1-14 days | 138 | 28 | 19.9 | 1.06 | (0.57-1.95) |
| Heart surgery risk | No influenza | 77 | 13 | 16.9 | 1.00 | (reference) |
|  | Influenza 1-14 days | 77 | 17 | 21.8 | 1.41 | (0.60-3.31) |
| Others surgery risk | No influenza | 277 | 14 | 5.1 | 1.00 | (reference) |
|  | Influenza 1-14 days | 277 | 17 | 6.0 | 1.20 | (0.58-2.51) |
| General anesthesia | No influenza | 6809 | 673 | 9.9 | 1.00 | (reference) |
|  | Influenza 1-14 days | 6809 | 922 | 13.2 | 1.41 | (1.27-1.58) |
| No general anesthesia | No influenza | 3463 | 252 | 7.3 | 1.00 | (reference) |
|  | Influenza 1-14 days | 3463 | 366 | 10.3 | 1.50 | (1.26-1.79) |
| CI, confidence interval; OR, odds ratio.  *Adverse events included with pneumonia, septicemia, acute renal failure, urinary tract infection.  †Crude odds ratio after propensity-score matching | | | | | | |
